# Effects of elevated mean and extremely high temperatures on the physio-ecological characteristics of geographically distinctive populations of *Cunninghamia lanceolata*

**DOI:** 10.1038/srep39187

**Published:** 2016-12-22

**Authors:** Ting Zhou, Xiaorong Jia, Huixuan Liao, Shijia Peng, Shaolin Peng

**Affiliations:** 1 State Key Laboratory of Biocontrol, School of Life Sciences, Sun Yat-sen University, Guangzhou, 510006, China; 2 College of Forestry and Landscape Architecture, South China Agricultural University, Guangzhou, 510642, China

## Abstract

Conventional models for predicting species distribution under global warming scenarios often treat one species as a homogeneous whole. In the present study, we selected *Cunninghamia lanceolata (C. lanceolata*), a widely distributed species in China, to investigate the physio-ecological responses of five populations under different temperature regimes. The results demonstrate that increased mean temperatures induce increased growth performance among northern populations, which exhibited the greatest germination capacity and largest increase in the overlap between the growth curve and the monthly average temperature. However,tolerance of the southern population to extremely high temperatures was stronger than among the population from the northern region,shown by the best growth and the most stable photosynthetic system of the southern population under extremely high temperature. This result indicates that the growth advantage among northern populations due to increased mean temperatures may be weakened by lower tolerance to extremely high temperatures. This finding is antithetical to the predicted results. The theoretical coupling model constructed here illustrates that the difference in growth between populations at high and low latitudes and altitudes under global warming will decrease because of the frequent occurrence of extremely high temperatures.

Under climate change, shifting species distribution has a direct impact on biodiversity and ecosystem function^1^. Temperature is one of the key environmental factors that dictates the growth and distribution of species. Consequently, a change in temperature will have a profound impact on individuals, populations and communities, even on the structure and function of the natural ecosystem^2^. Generally, there are four possible scenarios of plant distribution change under global warming: extinction, reduction of the distribution area, expansion of the distribution area and migration. In the first scenario, the species becomes extinct because it fails to adapt to temperature increases^3^. For example, Feeley *et al*. predicted that if the pace of climate warming exceeds a species’ ability to migrate^4^, all of the plant species in the Andeans will experience large population losses and consequently face a high risk of extinction. However, temperature increases will not have an extreme impact on all species. A second scenario may occur:when species are unable to adapt to the high temperatures at the edges of their distribution areas, the distribution areas will shrink^3^,^5^,^6^. Zhu *et al*. found that the distribution areas of 58.7% of tree species examined in their study area decreased on both the northern and southern boundaries^5^. A third scenario may occur when a species positively responds to global warming^7^,^8^. Some studies have indicated that the abundance of shrubs in frigid or near-frigid zones has increased in recent years^7^,^8^. However, based on current findings, the occurrence of the fourth scenario is more prevalent: temperature increases will result in a shift in the distribution area of a species^9^,^10^, including shifts in the distribution boundary and distribution center.

As a result of temperature increases, species have migrated toward nearby relatively low-temperature areas, i.e., areas at high latitudes and altitudes^11^,^12^,^13^. Song *et al*.^14^ studied six tree species on the Qinghai-Tibet Plateau and predicted that the distribution areas of five tree species would expand northward and westward, and that the distribution area of *Betula platyphylla* would migrate northward under future temperature increases. Honnay *et al*.^11^ also predicted that the distribution areas of approximately 80% of the forest species in Belgium would migrate northward by 2080. These models generally consider low temperatures in winter to be an important factor limiting the distribution areas of species. Therefore, temperature increases result in an increase in the duration of the growth periods of species in relatively frigid zones and can motivate these species to migrate toward areas that are originally even more frigid^15^. Although numerous models that are based on species characteristics and climate change predict that global warming will result in species extinction and a decrease in species distribution areas^1^,^16^, actual observation results are complex and varied; the distribution areas may decrease or increase due to global warming.

When predicting the distribution area of a certain species using conventional models, all of the populations of this species are treated as a homogeneous whole, and predictions are made based on three cardinal points of temperature and thermal tolerance of the species. There have been few studies of different populations of the same species and their response to global warming. These models fail to take into account the difference among populations in different distribution regions. Additionally,extensive evidence obtained in biogeographical research shows that populations will become, to a certain extent, adapted to the environmental conditions of their habitats^17^,^18^,^19^,^20^. According to the hypothesis of natural selection, due to the selection by the natural environmental conditions of different distribution regions, different populations of the same species may evolve to have characteristics that are most suitable for distribution in regions they inhabit. Due to geographical isolation and the lack of a suitable local habitat, a decrease in the frequency of mating between plant individuals with increased spacing distance, and the isolation of populations caused by the spatial effectiveness of the dispersion of plant progeny, are the results of the natural selection of organisms by the local habitat, which may consequently result in differentiation in the responses of populations in different regions or different individuals in the same region to ecological factors^21^. This differentiation includes differentiation in the temperature niches and thermal tolerance of high temperatures. Because there may be a difference in the characteristics among populations in different distribution regions, and because the environmental conditions of different distribution regions may undergo different changes under global climate change, it is very important to discriminate different populations of a species when studying the impact of global climate change on species distribution. In doing so, the change in the distribution area of the species under global warming can be more comprehensively and accurately predicted^22^.

For any arbitrarily dispersed species distributed over a large area, we can infer that there is likely differentiation in the three cardinal points of temperature and thermal tolerance of different populations of this species; i.e., the lowest, optimal and highest temperatures suitable for the growth of populations in different regions are not entirely consistent with each other. Generally, increases in global average temperature are beneficial for the growth and physiological metabolism of plants. When the temperature increases to extremely high temperatures, plants may suffer from many negative effects due to heat stress. For example, seed germination rates and seedling growth increase rapidly with increasing temperatures within a certain range; however, when the temperature rises to an extreme level, seed germination decreases significantly^23^. In terms of photosynthesis, there is a significant positive relationship between photosynthesis of *Pinus taeda* and temperature increases^24^. The response of net photosynthetic rates of *C. lanceolata* leaf to increasing temperature is parabolic^25^. Moreover, under high temperature stress, the chlorophyll content of most rice varieties exhibited a downward trend^26^. With continuously increasing global temperatures, particularly the frequent occurrence of extremely high temperatures, the responses of different populations to temperature should be different. For example, Calosi *et al*.^27^ found that populations at low latitudes and altitudes can better respond to global warming because of their higher tolerance to high temperatures and can thus benefit from global warming, whereas populations at high latitudes and altitudes fall victim tothe extremes of global warming because of their inability to adapt to high-temperature environments. However, it is necessary to note that even for a population that has adapted to a high-temperature environment, if the temperature increases above the upper-limit temperature of this population, a decrease in the local population abundance will occur because this population is unable to adapt to high temperatures, which in turn results in a change in the distribution area of the species^28^,^29^,^30^. Although this change in the distribution area is consistent with the results obtained using conventional prediction models that treat a species as a homogeneous population, the mechanisms are different. Therefore, by simultaneously comparing the response of different populations of one dispersed species to global change, we are likely to obtain conclusions that are more accurate than those obtainedfrom conventional evaluation models.

Based on the above theory, we predict that the increase in future average temperatures will have promoting effects among northern populations by prolonging their growing season. When the temperature reaches extreme heat levels, northern populations willbe negatively affected because they are unable to endure high temperature stress. Conversely, southern populations may not be negatively affected by heat stress due to their evolved heat adaptability when growing in a hot environment for a long time. We directly used a fitness related index, including colonization variables (e.g., germination rate, survival) and growth- or competition-related variables (e.g., biomass), to test this hypothesis. For the present study, we selected *C. lanceolata* as the experimental plant because of its large distribution area and wide temperature range, which can show different responses of species distributed in edge and center to the temperature change. We investigated the response patterns of the populations of *C. lanceolata* in different regions to temperature changes. Our goals were as follows: (1) to determine whether the temperature niches of different populations of the same species are different; (2) to determine how an elevated mean temperature affects the distribution areas of different populations; and (3) to evaluate the coupling effect of elevated mean temperature and extremely high temperatures on different populations.

## Materials and Methods

### Regions where the experimental material was collected

**Figure 1:**
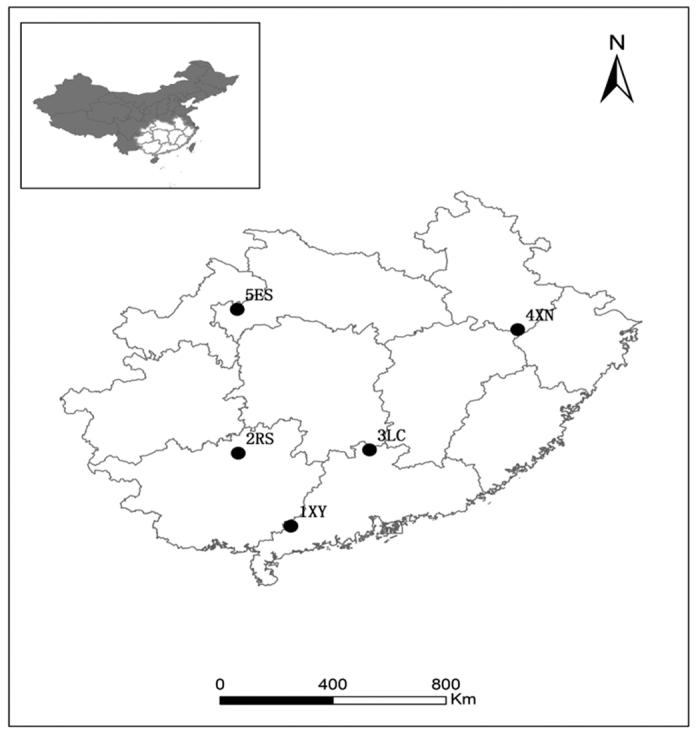
Sampling locations of the populations of *Cunninghamia lanceolate.* ES stands for Enshi, XN stands for Xiuning, LC stands for Lechang, RS stands for Rongshui, and XY stands for Xinyin. Figure 1 was drawn by Xiaorong Jia, and the maps here was generated by Arc GIS 9.0 (www.esri.com/software/arcgis).

*C. lanceolata* is an excellent fast-growing conifer species unique to southern China and is naturally distributed in the area between 101°13′ and 121°53′E and between 19°30′ and 34°03′N. The distribution area of *C. lanceolata* can be roughly divided into three regions: northern, central and southern, which correspond to the northern subtropical zone, the central subtropical zone and the southern subtropical zone, respectively. The central region (from northern Guangdong in the south to southern Anhui in the north) is considered to be the most suitable region for *C. lanceolata* growth. The relatively long dry period in the southern region (the edge of the southern region) and relatively low temperatures in winter in the northern region (the edge of the northern region) are important climate factors that restrict the development of populations of *C. lanceolata*^31^. Seeds of populations of *C. lanceolata* from the distribution area of *C. lanceolata* in China were collected from different latitudes in the field in the autumn and winter of 2012 as the experimental material (Fig. 1). For each seed collection area, seeds of 15–20 mother trees were collected and mixed in equal amounts and were used as the seeds representing the population of this area. The collected seeds were brought back to the laboratory and stored at a low temperature (4 °C).

The data for the annual average temperatures, average temperatures in January, average temperatures in July, annual average precipitation and sunshine hours in the five collection area between 1981 and 2010 were obtained from the China Meteorological Data Network (http://data.cma.gov.cn/) (Table 1).

### Seed germination experiment

The *C. lanceolata* seed germination experiment was conducted mainly using the method proposed by^32^,^33^. The seed germination experiment was carried out in an artificial climate chamber. Because the growth chambers available for our experiment were only four, we first conducted the experiment with each chamber corresponding with one temperature treatment for one month. Then we repeated the experiment with the same setting once. According to^34^, the extremely high temperature in the distribution area of *C. lanceolata* is 42 °C. Table 2 lists the temperature characteristics of each distribution region. Four different day/night temperature gradients were used in the experiment: 15 °C/10 °C, 22 °C/17 °C, 29 °C/24 °C and 35 °C/30 °C. These temperatures correspond to the annual average temperatures in the northern region, the annual average temperatures in the southern region, the monthly average temperatures during the hottest month in the distribution area, and the high temperatures in summer in the distribution area, respectively. The environment in an artificial climate chamber was set as follows: humidity, 80%; and Photosynthetic Photon Flux Density (PPFD), 200 μmol. m^−2^. s^−1^.

One hundred plump *C. lanceolata* seeds of approximately the same size that had been sterilized and disinfected were each placed into a petri dish (diameter = 9 cm). For each population of *C. lanceolata*, four replicates were performed for each temperature treatment.

### Seedling growth experiment

In June 2013, the germinated seeds were sown in plug trays (five seeds in each plug tray) and cultivated in an artificial climate chamber. After 22 days’ cultivation, the plug tray was changed to nutrition bag. One healthy seedling in each plug tray was transferred into 0.5 L pots with a substrate of 10:1 peat soil:perlite. During the seedling cultivation period, the nutrient solution was supplemented once every 10 d. The environment of the growth chamber was set as follows: temperature, 25/20 °C (12 h day/12 h night, the same below); humidity, 80%; and PPFD, 200 μmol· m^−2^·s^−1 ^35,36.

After five months of seedling cultivation, 20 *C. lanceolata* seedlings of similar size were selected and placed in four artificial climate chambers, corresponding to the four different day/night temperature gradients used in the seed germination experiment.

Eight seedlings from each population were randomly selected before the temperature treatments and subjected to testing to determine the total biomass of each plant and its distribution as well as the dry weight (W1). One month later, eight healthy seedlings were selected from each treatment and each population. Each of these seedlings was removed whole from the substrate and cleaned. Then, each seedling was divided into three parts: roots, stems and leaves. The fresh weight of each part was determined. The plant materials were dried at 80 °C until there was no further weight loss. The dry weight of each tissue was determined. Based on the aforementioned measurements, the total dry weight (W2) and its allocation (proportions of roots, stems and leaves of the total biomass of the plant), the root-shoot ratio, and the relative growth rate (RGR) were calculated.

The RGR is calculated using the following equation:


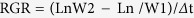


where W1 and W2 represent the biomass at the first sampling time and the second sampling time, respectively, and Δt represents the time interval between the first sampling time and the second sampling time. The RGR unit is in g·g^−1^·d^−1^. In addition to biomass, we also determined the chlorophyll content of the leaves, the photosynthetic efficiency of the leaves, and the chemical composition of the leaves. The chlorophyll in the leaves was extracted using the ethanol extraction method^37^. The indexes for evaluating the photosynthetic efficiency of the leaves mainly include the maximum photosystem II (PS II) light energy conversion efficiency (variable fluorescence (Fv)/maximum fluorescence (Fm)), PS II electron transport (Fv/initial fluorescence (F_0_)), and actual PS II photosynthetic efficiency (Y(II)). The required parameters were measured using a PAM-2500 portable pulse-amplitude modulation fluorometer (Walz, Germany). The indices for determining the chemical composition of the leaves include non-structural carbohydrates (i.e., soluble sugars and starch) and the C, N and P contents of the leaves. The soluble sugar and starch contents of each organ of each plant were determined using the anthrone-sulfuric acid colorimetric method^38^. The C content of the leaves was determined using the potassium dichromate-concentrated sulfuric acid oxidation method with external heating^39^. Prior to the determination of the N and P contents of the leaves, the leaves were subjected to a sulfuric acid-hydrogen peroxide digestion process. Afterward, the N content of the leaves was determined using a Kjeltec N analyzer (KDN-102F, Shanghai Xianjian Instruments CO., LTD), and the P content of the leaves was determined using an ultraviolet spectrophotometer^39^.

### Data analysis

Using the general linear model, we compared the different populations in terms of their seed germination and seedling growth responses to different temperature treatments. In these models, population, temperature and population-temperature interaction were selected as the explanatory factors, whereas germination percentage, biomass and RGR were the dependent factors.

By fitting the RGRs of each population under different temperature treatment conditions (n = 8 for each treatment) and temperature into a quadratic function, a quadratic function of the RGR of each population with respect to temperature was obtained. Based on the obtained function, we calculated the optimal temperature for the growth of each population (the temperature at which RGR was the highest) and the temperature at which the growth rate of each population was zero. Thus, the temperature niche for the growth of each population was obtained. Furthermore, to quantify the fitness of each population under the current temperature conditions and the temperature conditions after a temperature increase of 3 °C, we calculated the area of the quadratic RGR-temperature curve that overlaps the current temperature range and the temperature range after the temperature increased as well as the proportion of the overlapped area from the total area within the temperature niche of the population, which was then used as the standardized overlapped area.

Furthermore, to thoroughly compare the stability of the response of the populations in the northern and southern regions to the temperature treatments to characterize the difference in tolerance among the different populations, we extracted the data of the population from Enshi, Hubei in the northern region and the population from Xinyi, Guangdong in the southern region for analysis. The main indices of the two compared populations included the germination percentage, the biomass, the chlorophyll content, the photosynthetic efficiency, and the stability of the chemical composition of the leaves under the annual average temperature (Enshi: 15 °C; Xinyi: 22 °C), increased temperature (29 °C) and extremely high temperature (35 °C) conditions of the originating region. For each index, we selected the population (Enshi versus Xinyi), the temperature treatment (optimal temperature, increased temperature and extremely high temperature) and the population-temperature interaction as the fixed factors of the general linear model. A significant population-temperature interaction indicates that there was a difference in the stability of the response to a temperature increase between the population from Enshi in the northern region and the population from Xinyi in the southern region. A more stable response indicates greater tolerance to temperature increases.

## Results

### Germination response of different populations of *C. lanceolata* to temperature

**Figure 2:**
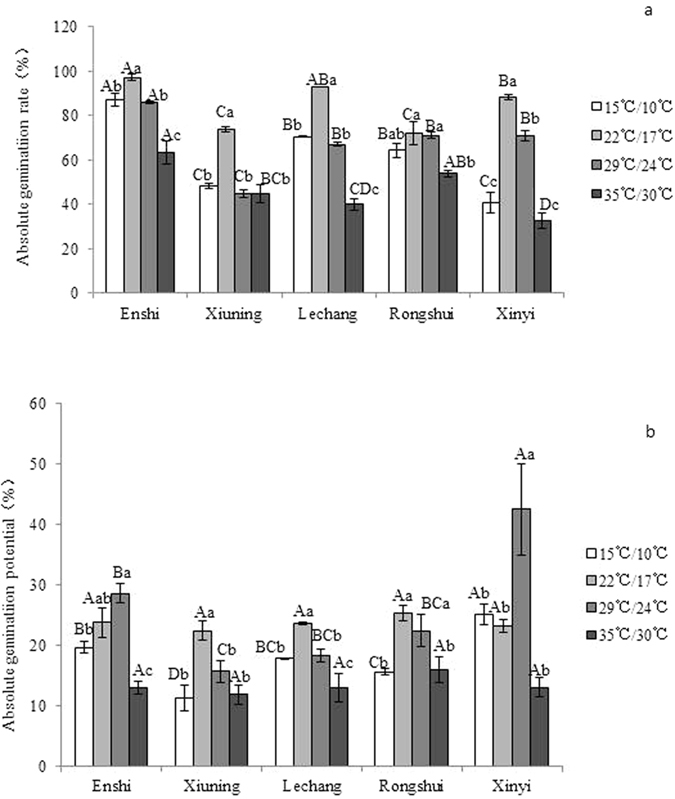
Response of the germination percentages of the seeds of different populations of *C. lanceolata* to temperature. The data are expressed as average valuesLeaf starch (mg·g^−1^) standard deviations. The lowercase letters indicate the analysis of the difference within the same population under different temperature treatments. The uppercase letters indicate the analysis of the difference among different populations under the same temperature treatment (P < 0.05).

Temperature, population origin and temperature-population interaction all had significant effects on the germination percentage (Table 3). Of the four temperature treatments, the germination percentage of each of the five populations reached its maximum under the 22 °C/17 °C temperature treatment. Compared with other populations, the germination percentage of the population from Enshi in the northern region was greatest under all the temperature treatments (Fig. 2). At 15 °C/10 °C and 29 °C/24 °C, the absolute germination potentialof the population from Xinyi in the southern region was higher than that ofthe other populations (Fig. 2) (P < 0.05), indicating that the populations in the northern region had the greatest germination capacity and that the population from the southern region had the fastest germination speed.

### Biomass and RGR responses of the seedlings of different populations of *C. lanceolata* to temperature

**Figure 3:**
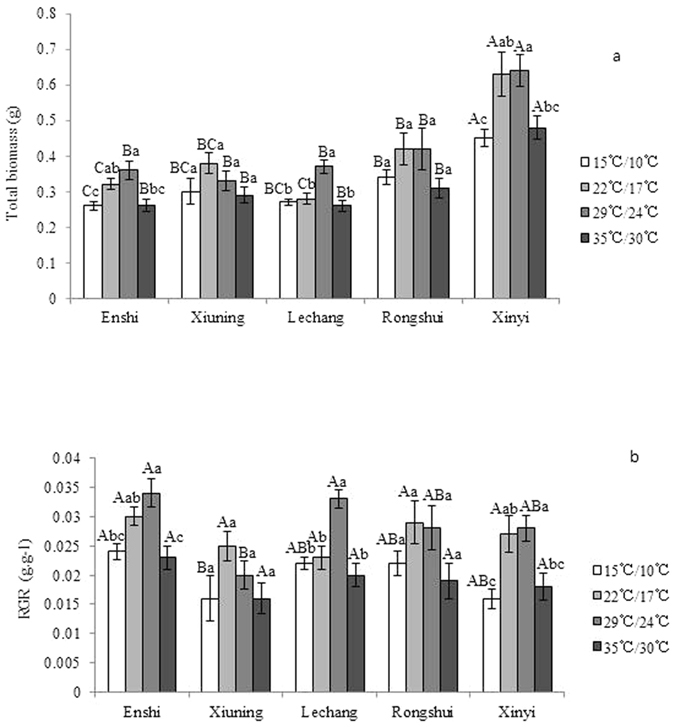
Response of the biomass and RGRs of the seedlings of different populations of *Cunninghamia lanceolata* to temperature. The data are expressed as average values ± standard deviations. The lowercase letters indicate the analysis of the difference within the same population under different temperature treatments. The uppercase letters indicate the analysis of the difference among different populations under the same temperature treatment (P < 0.05).

Temperature and the population origin had a significant impact on the growth and RGR of the seedlings, whereas the temperature-population interaction had no significant impact on the growth and RGR of the seedlings (Table 3). The biomass of seedlings from Xinyi in the southern region was the largest compared with the other populations under any temperature treatment, followed by the population from Rongshui in the central region (Fig. 3). The biomass decreased significantly when the temperature was excessively low or high. The effects of the four temperature treatments on the RGRs of the five populations exhibited a similar pattern (Fig. 3).

### Overlapped areas between the growth curves and the monthly average temperature ranges of different populations of *C. lanceolata*

**Figure 4:**
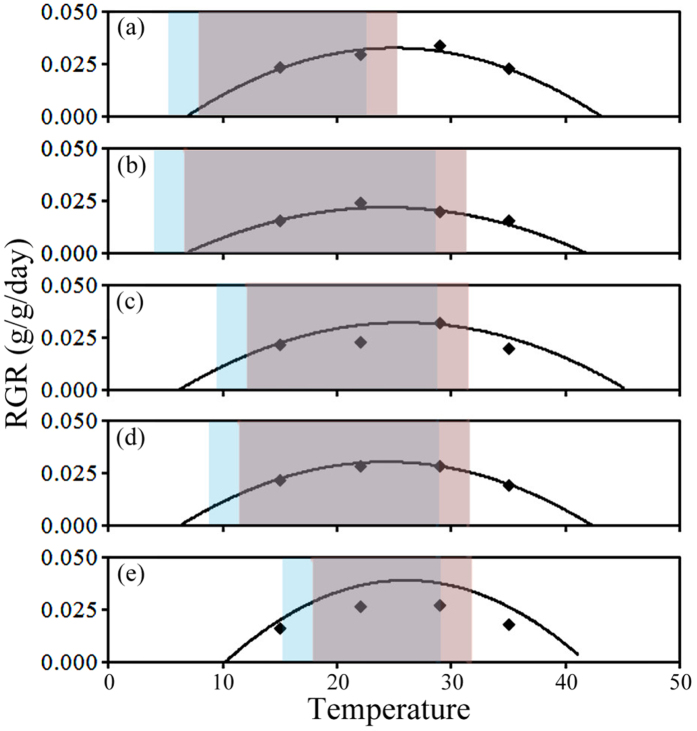
Environmental temperature ranges, current monthly average temperature variation ranges, and monthly average temperature variation ranges after a temperature increase of 3 °C of the five populations. (**a**) Enshi, (**b**) Xiuning, (**c**) Lechang, (**d**) Rongshui, and (**e**) Xinyin. The curves shown in the figure are quadratic RGR-temperature function regression curves (Enshi: y  = −0.0001x^2^ + 0.005x − 0.0297 [R^2^ = 0.40, P = 0.001]; Xiuning: y = −0.00008x^2^ + 0.0038x − 0.0227 [R^2^ = 0.16, P = 0.081]; Lechang: y = −0.00007x^2^ + 0.0038x − 0.0198 [R^2^ = 0.24, P = 0.017]; Rongshui: y = −0.00009x^2^ + 0.0044x − 0.0235 [R^2^ = 0.18, P = 0.051]; Xinyi: y = −0.0001x^2^ + 0.0057x − 0.0438 [R^2^ = 0.38, P = 0.001]). The blue areas indicate the current monthly average temperature variation ranges of the provenances of the populations. The red areas indicate the monthly average temperature variation ranges of the provenances of the populations after a temperature increase of 3 °C.

The environmental temperature of the population from Xinyi in the southern region ranged from 9.5 °C to 41.6 °C, with a range of 32.1 °C (Fig. 4). The environmental temperature of the population from Lechang in the central region ranged from 5.8 °C to 45.1 °C, with a range of 39.3 °C. The environmental temperature of the population from Rongshui in the central region ranged from 6.2 °C to 42.5 °C, with a range of 36.3 °C (Fig. 4). The environmental temperature of the population from Enshi in the northern region ranged from 6.9 °C to 43.6 °C, with a range of 36.7 °C and the environmental temperature of the population from Xiuning in the northern region ranged from 7.0 °C to 42.0 °C, with a range of 35.0 °C (Fig. 4).

Under the current temperature conditions, the order of overlapped area sizes between the growth curves and the monthly average temperature ranges of the populations were as follows (from largest to smallest): Rongshui in the central region (65%), Xiuning in the northern region (64%), Lechang in the central region (58%), Xinyi in the southern region (56%) and Enshi in the northern region (40%) (Fig. 4). After the average temperature increased by 3 °C, the overlapped areas between the growth curves and the monthly average temperature ranges of the population from Enshi and the population from Xiuning in the northern region increased to the largest degree, to 51% (an 11% increase) and 76% (a 12% increase), respectively (Fig. 4). Furthermore, the overlapped areas of the populations from Rongshui and Lechang in the central region increased to various degrees, to 71% (a 6% increase) and 64% (a 6% increase), respectively (Fig. 4). The overlapped area of the populations from Xinyi in the southern region increased to 60% (a mere 4% increase).

### Tolerance of the populations in the northern and southern regions to temperature increases

In terms of the response of seed germination to temperature, the population from Enshi in the northern region outperformedand was more stable than the population from Xinyi in the southern region after the temperature increased (Tables 4 and 5). In terms of the response of seedling growth to temperature, the individual seedlings of the population from Xinyioutperformed those of the population from Enshi, but there was no difference in the stability of the response of seedling growth to temperature increase between these two populations (Tables 4 and 5). In terms of photosynthetic pigments, the total chlorophyll content of the southern region population was significantly greater than that of the population from the northern region, and the response of chlorophyll content to temperature increase in the southern region population was more stable than that of the population in the northern region. There was no difference in the stability of chlorophyll a and b between the populations in the southern and northern regions (Tables 5 and 6). In terms of photosynthesis, the population from the southern region outperformed the population from the northern region in maximum PS II light energy conversion efficiency (Fv/Fm) and electron transfer (Fv/F_0_), and the population from the southern region was also more stable than the population from the northern region (Tables 5 and 6). There was no significant difference in the actual PS II photosynthetic efficiency between the populations in the northern and southern regions, but the population from the northern region was more stable than the population from the southern region (Tables 5 and 6). Regarding the chemical composition of the leaves, the soluble sugar content of the leaves of the northern region population was greater than that of the population from the southern region, and the soluble sugar content response of the leaves of the population from the northern region to temperature was also more stable compared with the population from the southern region (Tables 5 and 6). However, the C and N contents of the leaves of the southern region population were greater than those of the northern region population, and the response of the C and N contents of the leaves to temperature in the southern region population was more stable than that in the northern region population (Tables 5 and 6). The P content of the leaves in the northern region population was greater than the southern region population, whereas the response of the P content of the leaves to temperature in the southern region population was more stable than that of the northern region population (Tables 5 and 6). In summary, of the 13 indexes determined in the present study, the response of seven indices to temperature in the southern region population was more stable compared with the northern region population; there was no significant difference in the stability of the responses of three indices to temperature between the populations in the southern and northern regions; and the responses of three indices to temperature in the northern region population were more stable compared with the southern region population.

## Discussion

Differences in the responses to temperature increases not only exist among different species but also between different populations of the same species. Generally, studies of the change in the distribution area of a species under global change consider the study area species as a homogeneous whole and do not differentiate between different populations of the species^1^,^16^,^40^. The present study determined that there is a difference in the response to temperature change among populations of *C. lanceolate* collected from different regions (Figs 2 and 3); the influence of temperature increases on the growth of the populations exhibited an increasing trend with increasing latitude (Fig. 4). Therefore, we predicted that the populations of *C. lanceolata* in different regions would all respond by growing faster under temperature increases, and we also predicted that the influence on the growth of northern region populations would be relatively more prominent than those from the southern region.

The various responses to temperature among different populations resulted in a complex migration pattern in the distribution of this species. In a scenario in which the global temperatures increase by 3 °C, the population from the southern region may be less likely to expand its range, whereas the populations in the northern region are very likely to expand northward, which is basically consistent with the results of the predictions for the distribution of *C. lanceolate* based on the current suitable habitats of *C. lanceolata*^40^,^41^, i.e., the northern edge of the distribution area of *C. lanceolata* in China will expand northward. However, some models predict that the central region will become the most suitable area for the growth of populations of *C. lanceolata* under global climate change^40^,^41^. In the models of Liu *et al*.^41^ and Lu *et al*.^40^ although the southern edge of the distribution area did not move northward, the growth of the population from the southern region was significantly inhibited, which is different from the results of the experiments conducted based on the response to temperature change for the populations in different regions in the present study. We believe that temperature increases will not significantly inhibit the growth of the population from Xinyi in the southern region and that the area most suitable for the growth of *C. lanceolata* will shift from the central region to the northern region. These two models were both established based on the current distribution area of *C. lanceolata* in China, which assumed that the current distribution area of *C. lanceolata* completely reflects the range of the environmental conditions to which the species of *C. lanceolata* in China has adapted and that the temperature range and the water content range suitable for growth of all the populations of *C. lanceolata* are the same. When the differentiation of the temperature niche of different populations is not taken into account, it can be easily concluded that the central region is the most suitable area for the growth of *C. lanceolata* because the central region currently has the intermediate climate conditions of the whole distribution area of *C. lanceolata.* It is very likely that homogenization of the populations of *C. lanceolata* is the main reason for the contradiction between the predictions obtained using these models and our results. Because of the difference in the climate conditions among the habitats where different populations grow and the difference in the adaptability to extremely high temperatures among different populations, there is a difference in the response to temperature among different populations.

**Figure 5:**
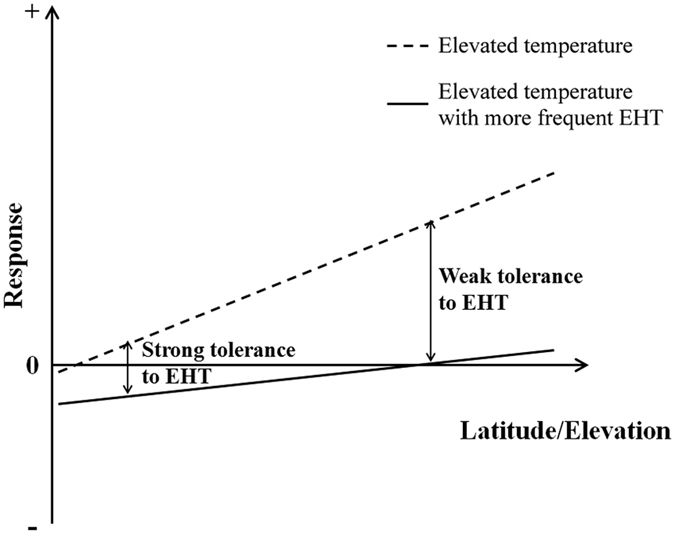
Pattern of the responses of different populations of the same species in different latitudes/altitudes to an average temperature increase and an extremely high temperature and the coupling effect of the average temperature increase and the extremely high temperature. The dotted line indicates a scenario in which the average temperature increases. The solid line indicates a scenario in which the average temperature increases, which is also accompanied by an increase in the frequency of the occurrence of extremely high temperatures. With greater latitude and altitude of the provenance of the population, the promoting effect of the temperature increase on the growth of the population becomes stronger, and the tolerance of the population to extremely high temperature stress may become weaker. Therefore, the advantages of populations in high latitudes/altitudes will decrease under extremely high temperature conditions.

In fact, in addition to the increase in average temperatures, global warming is also characterized by an increase in the frequency of the occurrence of extremely high and low temperatures^42^,^43^. Extremely high and low temperatures have a coupling effect on plants. Based on meteorological data, we know that, except for in Enshi, Hubei, there was no significant difference in the average temperature of the hottest month among the other regions (Fig. 4), i.e., the frequencies with which the regions experience extremely high temperature events may be similar to each other. These results demonstrate that it is likely that predictions of the dynamics of these population distributions based solely on the temperature range for each population of *C. lanceolata* cannot reflect the future distribution of *C. lanceolata.* Based on the response of different populations of *C. lanceolata* to an average temperature increase and extremely high temperatures, we predict that under future temperature increases, whereas the southward expansion of the southern edge of the distribution area of *C. lanceolata* is unlikely, the growth of the population of *C. lanceolata* in Xinyi in the southern region will not be significantly inhibited. This prediction is different from the results obtained using previous models. On account of its long-term adaptation to climate conditions in the southern region, the population from the southern region should be the most stable in a scenario in which the temperature increases and extremely high temperatures occur (Fig. 5). Therefore, we believe that the populations of *C. lanceolata* in China should not be viewed as a homogeneous whole when predictinga change in the distribution area of *C. lanceolata* under global warmingconditions. However, it is worth noting that although the population from Xinyi in the southern region has no advantages over other populations exposed to extremely high temperature conditions, in terms of the seed germination percentage, these extremely high temperature events will not have a significantly negative impact on the population of *C. lanceolata* in Xinyi because natural germination mainly occurs in spring (Fig. 5).

It is common that different populations of dispersed species similar to *C. lanceolata* become adapted to local environments^44^,^45^. It is very important to differentiate between the different populations of a species when studying the impact of global warming on the distribution of the species^22^,^46^,^47^. Because of their differences in adaptability to temperature increases, the responses of the populations in different distribution regions to temperature increases will exhibit different characteristics^47^. Driscoll *et al*.^46^ analyzed the growth rings of *Picea glauca* in North America and found that the response of different populations of *P. glauca* to climate warming differed; whereas the individuals in the majority of the populations grew faster, the growth of the individuals in some populations was delayed. Similarly, our results show that the influence of temperature increases on the growth of the population increased with increasing latitude of the population’s region (Fig. 5). Furthermore, because average temperature increases are often accompanied by frequent occurrences of extremely high temperatures, the relatively low tolerance of populations in high latitudes/altitudes to extremely high temperatures may result in a significant decrease in the difference between populations at high latitudes/altitudes and populations at low latitudes/altitudes (Fig. 5). The durations of extremely high temperatures are relatively short, which may be the reason why there have been no studies into the effect of extremely high temperatures on species distribution. Because most previous studies into the effect of temperature increases on the distribution of a species fail to take into account the evolution different population adaptations to local climate conditions, these studies generally predict that temperature increases will have a more significant negative impact on populations at low latitudes and altitudes^9^,^48^. However, the difference among the regions along the change in latitude and altitude may be eliminated because of the adaptation of the populations to the local climate conditions, resulting in a discrepancy between the actual change in the distribution area of the species and the prediction made using a theoretical model. We believe that future models for predicting the distribution of species should take into account the adaptability of different populations to local and global climate conditions to more accurately predict the impact of global change on the species distributions.

## Additional Information

**How to cite this article**: Zhou, T. *et al*. Effects of elevated mean and extremely high temperatures on the physio-ecological characteristics of geographically distinctive populations of *Cunninghamia lanceolata. Sci. Rep.* **6**, 39187; doi: 10.1038/srep39187 (2016).

**Publisher's note:** Springer Nature remains neutral with regard to jurisdictional claims in published maps and institutional affiliations.

## Figures and Tables

**Table 1:** Sampling sites of *Cunninghamia lanceolata.*

Distribution region	Sampling sites	Lat. (°N)	Long. (°E)	Tmed (°C)	Tmin in Jan (°C)	Tmax in Jul (°C)	Rainfall (mm)	Annual solar radiation (hr)
Northern	Enshi, Hubei	30.34	109.25	16.3	5.2	22.6	1550	1267.9
	Xiuning, Anhui	29.60	118.18	16.5	4.2	27.9	1613.7	1931.4
Central	Lechang, Guangdong	25.20	113.46	19.8	9.6	28.2	1566.2	1728.7
	Rognshui, Guangxi	25.08	109.28	19.7	9	28.3	1878.3	1332.8
Southern	Xinyi, Guangodng	22.41	110.96	22.8	15.1	28.4	1760.1	1862.1

**Table 2:** Temperature characteristics of the distribution regions of *Cunninghamia lanceolata* 34.

	North-north subtropical zone	Mid subtropical zone	South -south subtropical zone	Suitablegrowth temperature
Annual average temperature	14–16 °C	16–21 °C	20–22 °C	16∼19 °C
Average temperatures in July	28 °C	29 °C	29 °C	
Average temperature in Suitable growth month				20–26 °C

**Table 3:** Results of two-way ANOVA of the effect of temperature, population and their interaction on germination and seedling growth of *Cunninghamia lanceolata.*

Trait	Temperatue		Population		Temperature × Population	
	F	P	F	P	F	P
Germination rate	163.12	<**0**.**001**	71.23	<**0**.**001**	2.84	<**0**.**001**
Total biomass	15.04	<**0**.**001**	46.07	<**0**.**001**	0.15	0.325
RGR	17.70	<**0**.**001**	6.43	<**0**.**001**	0.888	0.561

Significant results are shown in bold.

**Table 4:** The germination and growth performance of Enshi and Xinyi populations under favorable temperature, elevated temperature and extremely high temperature.

Population	T	Germination rate (%)		T	Total biomass (g)		T	RGR (g/g/day)	
		29 °C/24 °C	35 °C/30 °C		29 °C/24 °C	35 °C/30 °C		29 °C/24 °C	35 °C/30 °C
Enshi	87.12 ± 4.59	85.72 ± 0.90	63.29 ± 9.00	0.26 ± 0.03	0.36 ± 0.07	0.26 ± 0.05	0.02 ± 0.00	0.03 ± 0.01	0.02 ± 0.01
Xinyi	88.20 ± 2.25	70.83 ± 4.26	32.46 ± 5.85	0.63 ± 0.18	0.64 ± 0.13	0.48 ± 0.10	0.03 ± 0.01	0.03 ± 0.01	0.02 ± 0.01

**Table 5:** Results of two-way ANOVA of the effect of temperature, population and their interaction on the performance of *Cunninghamia lanceolata* (only Enshi and Xinyi populations were involved).

Trait	Temperatue		Population		Temperature×Population	
	F	P	F	P	F	P
Germination rate	97.11	**<0.001**	37.30	**<0.001**	14.29	**0.001**
Total biomass	6.29	**0.004**	95.67	**<0.001**	1.97	0.152
RGR	9.68	**<0.001**	1.87	0.179	2.33	0.109
Total chlorophyll	7.09	**0.009**	6.45	**0.026**	7.14	**0.009**
Chla/Chlb	3.90	**0.050**	9.93	**0.008**	2.69	0.109
Fv/Fm	3.48	**0.047**	9.39	**0.005**	16.97	**<0.001**
Fv/F0	1.58	0.227	9.83	**0.004**	15.65	**<0.001**
Y(II)	10.14	**0.001**	0.15	0.705	18.48	**<0.001**
Leaf soluble sugar	93.69	**<0.001**	21.71	**0.001**	12.47	**0.001**
Leaf starch	35.26	**<0.001**	0.62	0.447	2.21	0.152
Leaf C	6.79	**0.011**	44.17	**<0.001**	30.72	**<0.001**
Leaf N	280.70	**<0.001**	17.30	**0.001**	85.00	**<0.001**
Leaf P	393.70	**<0.001**	27.60	**<0.001**	258.70	**<0.001**

Significant results are shown in bold.

**Table 6:** The leaf physiology and chemical characteristics of Enshi and Xinyi populations under favorable temperature, elevated temperature and extremely high temperature.

Population	Chlorophyll (mg · g^−1^)			Chl a/Chl b			Fv/Fm			Fv/F0			Y(II) (mg · g^−1^)		
	T	29 °C/24 °C	35 °C/30 °C	T	29 °C/24 °C	35 °C/30 °C	T	29 °C/24 °C	35 °C/30 °C	T	29 °C/24 °C	35 °C/30 °C	T	29 °C/24 °C	35 °C/30 °C
Enshi	1.45 ± 0.33	1.62 ± 0.07	1.04 ± 0.03	3.75 ± 0.02	2.67 ± 0.28	3.39 ± 0.78	0.66 ± 0.04	0.76 ± 0.02	0.77 ± 0.02	2.01 ± 0.34	3.26 ± 0.37	3.36 ± 0.28	0.11 ± 0.02	0.13 ± 0.03	0.11 ± 0.01
Xinyi	1.80 ± 0.11	1.64 ± 0.33	1.62 ± 0.19	2.29 ± 0.26	2.55 ± 0.30	3.54 ± 0.29	0.79 ± 0.00	0.75 ± 0.05	0.76 ± 0.03	3.85 ± 0.12	3.10 ± 0.84	3.24 ± 0.48	0.17 ± 0.01	0.09 ± 0.02	0.09 ± 0.01
**Population**	**Leaf soluble sugar (mg · g^−1^)**			**Leaf starch (mg · g^−1^)**			**Leaf C (mg · g^−1^)**			**Leaf N (mg · g^−1^)**			**Leaf P (mg · g^−1^)**		
	**T**	**29 °C/24 °C**	**35 °C/30 °C**	**T**	**29 °C/24 °C**	**35 °C/30 °C**	**T**	**29 °C/24 °C**	**35 °C/30 °C**	**T**	**29 °C/24 °C**	**35 °C/30 °C**	**T**	**29 °C/24 °C**	**35 °C/30 °C**
Enshi	212.9 ± 11.8	206.3 ± 10.7	166.3 ± 5.5	34.7 ± 5.6	38.6 ± 0.8	20.7 ± 2.1	452.4 ± 8.2	470.7 ± 3.9	427.2 ± 6.3	35.1 ± 0.2	29.1 ± 0.4	40.0 ± 0.7	7.2 ± 0.1	4.6 ± 0.1	8.1 ± 0.2
Xinyi	214.2 ± 14.1	193.4 ± 2.3	112.0 ± 10.7	42.6 ± 5.7	40.0 ± 1.7	16.8 ± 8.2	478.7 ± 7.2	459.0 ± 7.2	477.7 ± 7.9	35.6 ± 0.6	34.2 ± 0.5	37.4 ± 0.5	6.0 ± 0.1	6.2 ± 0.1	6.8 ± 0.2
